# WHO Safe Surgery Checklist for Cesarean Section (CS) Delivery: Practice and Associated Factors Among Physicians in Rwanda

**DOI:** 10.1002/puh2.70112

**Published:** 2025-08-12

**Authors:** Rusagura Rwizigira Eric, Diomede Ntasumbumuyange, Abraham Fessehaye Sium

**Affiliations:** ^1^ Department of Obstetrics & Gynecology School of Medicine and Pharmacy College of Medicine and Health Science University of Rwanda Kigali Rwanda; ^2^ Department of Obstetrics and Gynecology St. Paul's Hospital Millennium Medical College (SPHMMC) Addis Ababa Ethiopia

**Keywords:** cesarean delivery, global health, public health, Rwanda, safe surgery, World Health Organization (WHO)

## Abstract

**Background:**

Cesarean delivery (CD) is a life‐saving intervention when vaginal delivery is not feasible. It is one of the most frequently performed procedures in obstetrics. This study examines the practice of adherence to the World Health Organization (WHO) safe surgery checklist for CD among doctors in Rwanda.

**Methods:**

A cross‐sectional survey was conducted among medical practitioners involved in Cesearan deliveries across Rwanda over 8 months in 2024. Data were collected on adherence to various pre‐, intra‐, and post‐operative protocols that stick to the WHO safe surgery checklist for cesarean section (CS) delivery. The data were collected prospectively using a pre‐tested questionnaire on Kobo Collect. Data were analyzed using STATA version 13 for analysis. Descriptive data are presented in frequencies and percentages in tables through univariate analysis. A *p* value ≤0.05 was considered statistically significant.

**Results:**

Significant gaps were found in adherence to pre‐operative protocols, with 78.26% of participants not conducting team handovers using the WHO surgical safety checklist. Additionally, 84.78% of practitioners did not ensure pre‐cesarean patient bathing with soap or the use of chlorhexidine, contrary to WHO recommendations. Intraoperative practices showed some alignment with the standards, such as the use of chlorhexidine for abdominal skin preparation (97.8%), but revealed deficiencies in thromboprophylaxis measures. Variability in operative techniques was noted, with 71.7% using the Pfannenstiel incision and the same percentage correctly placing the hysterotomy site.

**Conclusion:**

The study highlights critical gaps in adhering to pre‐operative, intra‐operative, and post‐operative WHO safe surgery checklist for Cesearan delivery in Rwanda. There is a need for improved training, adherence to standardized safety protocols, and enhanced post‐operative care practices to improve patient outcomes. Job title and clinical experience significantly influence adherence to best practices, underscoring the importance of specialized training.

## Graphical abstract

GRAPHICAL ABSTRACT

We found a high proportion of practitioners do not follow essential pre‐operative protocols, such as team handovers and the use of the WHO surgical safety checklist, and there is limited adherence to infection control practices like pre‐operative patient bathing with soap and vaginal preparation.

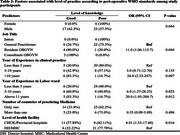

## 1 Introduction

Cesarean delivery (CD), defined as the delivery of a fetus via the abdomen following laparotomy and hysterotomy, is a critical life‐saving intervention when vaginal delivery is not feasible. It is one of the most frequently performed procedures in obstetrics [1]. However, cesarean sections (CSs) are associated with both immediate and long‐term complications [2]. Compared to spontaneous vaginal delivery, CSs are linked to higher rates of maternal and neonatal morbidity and mortality worldwide [3].

Major complications include postpartum endometritis, wound dehiscence/infection, hemorrhage, injury to pelvic organs, thromboembolic disorders, and anesthesia‐related complications [4]. Surgical site infections (SSIs) significantly contribute to morbidity and mortality, prolonging hospitalization and increasing the cost of patient care [5]. A study by Li et al. in China identified several independent risk factors for SSIs post‐CS: age ≥ 30 years (4.18%), BMI ≥ 24 (2.39%), duration of CS ≥ 1.5 h (1.2%), estimated blood loss ≥ 400 mL (2.35%), and urinary catheter duration ≥ 24 h [6]. In the United States, a randomized trial comparing antiseptic agents during CD (chlorhexidine‐alcohol vs. iodine‐alcohol) involved 1147 patients. Results showed that SSIs were diagnosed in 4.0% of the chlorhexidine‐alcohol group compared to 7.3% of the iodine‐alcohol group [7]. A study on the impact of surgical safety checklist (SSC) implementation on post‐cesarean sepsis revealed that higher SSC adherence correlated with lower maternal sepsis rates. The study concluded that SSC adherence could reduce maternal morbidity and enhance surgical quality interventions [8].

The World Health Organization's (WHO's) 2006 report on maternal mortality identified hemorrhage as the leading cause of maternal death globally. In developing regions, such as African and Asian countries, hemorrhage remained the primary cause, whereas in Latin America and the Caribbean, hypertensive disorders were the most prevalent [9]. In Rwanda, the 2021–2022 annual report on maternal and child mortality from the Rwanda Biomedical Center (RBC) indicated that out of 243 reported cases of maternal death, 82% were due to complications related to obstetric surgery (C‐section) and procedures [10]. The failure to adhere to CD guidelines and inadequate surgical skills are likely significant contributors to post‐CD complications in Rwanda. This study presents the practice of the WHO Safe Surgery Checklist for CS procedures among physicians in Rwanda.

## 2 Methods

### 2.1 Study Design, Study Setting, and Study Population

This was a prospective cross‐sectional study on the practice of the WHO safe surgery checklist for CD among physicians who practice CD in Rwanda. The study was conducted across 12 healthcare facilities in Rwanda, encompassing medicalized health centers, district hospitals, provincial hospitals, and referral hospitals, over a period of 8 months (January 2024 to August 2024). The participating teaching hospital was Kigali University Teaching Hospital. Provincial hospitals involved in the study were Rwamagana, Ruhango, Kinihira, and Bushenge Provincial Hospitals. District hospitals spanned multiple provinces: in the Eastern Province, Kirehe District Hospital was included; in the Southern Province, Kabutare District Hospital was selected; in the Northern Province, Nemba District Hospital was selected; in the Western Province, Gisenyi District Hospital was selected; and in Kigali City, Muhima and Kibagabaga District Hospitals. Additionally, the study included the medicalized health center of Rutare. The study population consisted of intern doctors, general practitioners, and residents in obstetrics and gynecology, and consultants in obstetrics and gynecology who performed CSs at their respective health facilities.

### 2.2 Sample Size and Sampling Procedure

We considered 95% confidence interval, margin of error of 5%, and *p* value (the estimated proportion of medical doctors with adherence to WHO standards) to be 50%, as there is no previously known proportion from local studies. The calculated minimum required sample from our population was 45 participants, and we managed to recruit 46 participants. From each province, secondary‐level health facilities were categorized into strata. Within each stratum, including those in Kigali City, two hospitals were randomly selected. At least four medical doctors were recruited from each selected health facility, except for Rutare Medicalized Health Center, where only two doctors were recruited.

### 2.3 Inclusion Criteria and Exclusion Criteria

Inclusion criteria:

- being a medical doctor, namely, intern doctors, general practitioners, resident in obstetrics, obstetrician, and gynecologist currently practicing in a selected facility;
- working in gynecology and obstetrics department and performing CDs.

Exclusion criteria

- medical doctors who did not want to be part of the study were excluded.

### 2.4 Data Collection Procedures

Data were collected from intern doctors, general practitioners, residents, and gynecologists using a structured questionnaire designed in Kobo Collect v2022.4.4, available in both mobile applications and web‐based versions. The questionnaire included a Likert scale ranging from 0% to 100% to capture responses. Hospital and medicalized health center directors were informed about the study 1 month in advance.

The study began with the identification of health facilities and participants. A research assistant was stationed at each hospital to collect data from identified and consented participants. The researcher first explained the purpose of the research and confidentiality to the participants. Data were gathered through observations of performance of CS using the structured questionnaire, allowing the researcher to explore various areas of inquiry. Participants received detailed information about studying the local language before giving their consent. Both verbal and written consents were obtained prior to the interviews. Prior to data collection, a pilot study was conducted with the data collectors to ensure the tools’ comprehensibility. A 1‐day pilot study was carried out 1 week before the main study at a district hospital similar to the study setting. The tools were tested on five participants. Subsequently, the tools were adjusted and improved on the basis of feedback and observations from the pilot study.

### 2.5 Data Analysis

After gathering information from participants using Kobo Collect, all data were transferred to STATA version 13 for analysis. Descriptive data are presented in frequencies and percentages in tables through univariate analysis. The association between dependent and independent variables within the group was determined using the chi‐square test and logistic regression. A *p* value ≤0.05 was considered statistically significant.

## 3 Results

Among the study participants, 86.96% are males and 13.04% are females (Table 1). In terms of job titles, 65.22% are general practitioners, 13.4% are intern doctors, 10.87% are residents, and 10.87% are obstetricians and gynecologists. Regarding their experience in clinical practice, 54.35% have less than 5 years of experience, 30.43% have 5–10 years, 13.04% have 10–15 years, and 2.17% have over 16 years. In labor and delivery care, 65.22% have less than 5 years of experience, 21.74% have 5–10 years, and 13.04% have 10–15 years. Lastly, 80.43% practice medicine in only one country, 17.39% in two countries, and 2.17% in three countries.

**TABLE 1:** Sociodemographic characteristics of physicians who practice Cesarean section procedures in Rwanda, 2023.

Characteristics	*n*	%
**Gender**		
Female	6	13.04
Male	40	86.96
**Job tittle**		
Intern doctor	6	13.4
General practitioner	30	65.22
Resident	5	10.87
Obstetrician and gynecologist	5	10.87
**Experience in working in clinical practice (years)**		
<5	25	54.35
5–10	14	30.43
10–15	6	13.04
>16	1	2.17
**Experience in working in labor and delivery care (years)**		
<5	30	65.22
5–10	10	21.74
10–15	6	13.04
**Number of countries of practicing medicine**		
Only one	37	80.43
Two	8	17.39
Three	1	2.17

A majority, 78.26%, do not conduct pre‐CS team handovers, and 73.91% do not routinely perform obstetric handovers (Table 2). Similarly, 78.26% are not familiar with the WHO SSC, and the same percentage lack training in evidence‐based clinical practice protocols for CSs. However, 60.87% possess adequate physician or assistant surgical knowledge for CSs. Only 23.91% have received surgical team training, whereas a significant 84.78% do not ensure pre‐CS patient bathing with soap, and none use chlorhexidine soap. Encouragingly, 76.09% administer prophylactic antibiotics within 60 min before starting a CS, indicating some adherence to pre‐operative protocols. Almost all practitioners (97.8%) do not perform intraoperative hair removal, but an equal percentage (97.8%) (Table 3) do use chlorhexidine for abdominal skin preparation, and 80.43% use povidone iodine for CSs. The majority of medical doctors (71.7%) use the Pfannenstiel skin incision, whereas 95.6% do not create a bladder flap prior to hysterotomy. Similarly, 71.7% make the hysterotomy site approximately 6 cm above the pubic bone, and 95.6% extend the hysterotomy bluntly with fingers rather than sharply with scissors. For placental delivery, 71.74% use controlled cord traction followed by a manual sweep, whereas 28.3% use controlled cord traction without a manual sweep. Additionally, 97.8% practice double‐layered closure of the hysterotomy, contrasting with only 2.2% who use single‐layered closure. The parietal peritoneum is not closed by 89.1% of practitioners, and 86.9% close the subcutaneous tissue if it is 2 cm in depth. A significant majority (91.3%) of medical doctors remove the incisional dressing 48 h after surgery and also remove the Foley catheter immediately or within 1 day post‐surgery.

**TABLE 2:** Practice of pre‐, intra‐, and post‐operative World Health Organization (WHO) safe surgery checklist for cesarean section among physicians in Rwanda.

Practice of C/S pre‐operative WHO standards	*N*	%
**Pre‐caesarean section team handover**		
No	36	78.26
Yes	10	21.74
**Routine obstetric handover**		
No	34	73.91
Yes	12	26.09
**WHO surgical safety check list**		
No	36	78.26
Yes	10	21.74
**Training in evidence‐based clinical practice protocol of cesarean section**		
No	36	78.26
Yes	10	21.74
**Physician/Assistant surgical knowledge in cesarean section**		
No	18	39.13
Yes	28	60.37
**Surgical team training**		
No	35	76.09
Yes	11	23,91
**Pre‐cesarean section patient bathing with soap**		
No	39	84.78
Yes	7	15.22
**Pre‐cesarean section patient bathing with chlorhexidine soap**		
No	46	100
Yes	0	0
**Prophylactic antibiotics within 60 min of start of cesarean section**		
No	11	23.91
Yes	35	76.09
**Intraoperative hair removal**		
No	45	97.83
Yes	1	2.17
**Intraoperative abdominal skin preparation with chlorhexidine**		
No	1	2.17
Yes	45	97.83
**Intraoperative abdominal skin preparation with povidone iodine for C/S**		
No	9	19.57
Yes	37	80,43
**Intraoperative vaginal preparation in the operating room**		
No	34	73.91
Yes	12	26.09
**Routine administration of oxytocin or carbetocin at C/S time**		
No	1	2.17
Yes	45	97.83
**Administration of additional medications (other than oxytocin/carbetocin) to treat PPH**		
No	31	67.39
Yes	15	32.61
**Placement of Foley catheter (bladder catheter) prior to C/S**		
No	2	4.35
Yes	44	95.65
**Use of headlamps during C/S, in case hospital loses electricity**		
No	46	100
Yes	0	0
**Use regional anesthesia for C/S**		
No	1	2.17
Yes	45	97.83
**Use general anesthesia for C/S**		
No	45	97.83
Yes	1	2.17
**Use of Joel Cohen skin incision for C/S**		
No	34	73.49
Yes	12	26.09
**Use of Pfannenstiel skin incision for C/S**		
No	13	28.26
Yes	33	71.74
**Use of bladder flap prior to hysterotomy**		
No	44	95.65
Yes	2	4.35
**Hysterotomy site made approx. 6 cm above pubic bone for C/S**		
No	13	28.26
Yes	33	71.74
**Hysterotomy extension bluntly with fingers for C/S**		
No	2	4.35
Yes	44	95.65
**Hysterotomy extension sharply with scissors for C/S**		
No	44	95.65
Yes	2	4.35
**Placental delivery with core traction followed by manual sweep**		
No	13	28.26
Yes	33	71.74
**Placental delivery with core traction without manual sweep**		
No	33	71.74
Yes	13	28.26
**Closure of hysterotomy with a double‐layered closure**		
No	1	2.17
Yes	45	97.83
**Closure of hysterotomy with a single‐layered closure**		
No	45	97.83
Yes	1	2.17
**Closure of parietal peritoneum**		
No	41	89.13
Yes	5	10.87
**Closure of subcutaneous tissue, if the tissue is 2 cm in depth**		
No	6	13.04
Yes	40	86.96
**Remove incisional dressing 48 h after C/S**		
No	4	8.7
Yes	42	91.3
**Inspect incision and examine abdomen daily during hospitalization and prior to discharge from hospital after C/S**		
No	23	50
Yes	23	50,00
**Foley catheter (bladder catheter) is removed immediately or 1 day after C/S**		
No	4	8.7
Yes	42	91.3
**Bladder catheter is removed on the day of discharge from hospital after C/S**		
No	42	91.3
Yes	4	8.7
**Foley catheter (bladder catheter) is kept in place after discharge following C/S**		
No	0	0
Yes	46	100
**Thromboprophylaxis using pneumonic compression devices during hospitalization for C/S**		
No	46	100
Yes	0	0
**Thromboprophylaxis using medications during hospitalization for C/S**		
No	39	84.78
Yes	7	15.22
**Follow an ERAS protocol for C/S**		
No	29	63.04
Yes	17	36.96
**Discharge instructions given regarding wound care at the time of discharge**		
No	26	56.52
Yes	20	43.48
**Patient instructed to have routine in‐person visit at the nearest hospital clinic following hospital discharge**		
No	35	76.09
Yes	11	23,91
**Patient called following hospital discharge for routine monitoring**		
No	45	97.83
Yes	1	2.17

**TABLE 3:** Factors associated with level of practice according to peri‐operative World Health Organization (WHO) standards among study participants.

Predictors	Level of knowledge		OR (95% CI)	*p* value
	Good	Poor		
Female	0 (0.0%)	6 (100%)		0.044
Male	17 (42.5%)	23 (57.5%)		
**Job title**				
Intern	0 (0.0%)	6 (100%)		
General practitioner	8 (26.7%)	22 (73.3%)	Ref.	
Resident OBGYN	4 (80.0%)	1 (20.0%)	11.0 (1.06–113.7)	0.044
Consultants OBGYN	5 (100%)	0 (0.0%)		
**Year of experience in clinical practice**				
Less than 5	5 (20.0%)	20 (80.0%)	Ref.	
5–10	6 (42.9%)	8 (57.1%)	3.0 (0.71–12.70)	0.135
>10	6 (83.3%)	1 (16.7%)	24.0 (2.33–247)	0.007
**Year of experience in labor ward**				
Less than 5	6 (20.0%)	24 (80.0%)	Ref.	
5–10	6 (60.0%)	4 (40.0%)	6.0 (1.27–28.25)	0.023
Above 11	5 (83.3%)	1 (16.7%)	20.0 (1.95–204.7)	0.012
**Number of countries of practicing Medicine**				
Only one	14 (33.8%)	23 (62.2%)	Ref.	
More than one	3 (25.0%)	6 (75.0%)	0.55 (0.09–3.09)	0.496
**Level of health facility**				
CHUK/Provincial hospitals	11 (57.89%)	8 (42.11%)	4.81 (1.33–17.40)	0.016
DH/MHC	6 (22.22%)	21 (77.78%)	Ref.	

Abbreviations: DH, district hospital; MHC, medicalized health center.

Job title is a significant predictor (Table 3), with residents in obstetrics and gynecology (OBGYN) and consultants in OBGYN showing higher levels of good practice compared to general practitioners, with residents being 11 times more likely to have a good level of practice compared to general practitioners (OR = 11.0, *p* = 0.044) and consultants having 100% good practice. Years of clinical practice experience indicate that those with more than 10 years of experience have significantly better practices, where they are 24 times more likely to have a good level of practice compared to those with experience less than 5 years (OR = 24.0, *p* = 0.007). Experience in the labor ward is also a significant predictor; practitioners with 5–10 years (OR = 6.0, *p* = 0.023) and above 11 years (OR = 20, *p* = 0.012) of experience in the labor ward have higher odds of good practice compared to those with <5 years of experience. Working area/Hospital was associated with good practice, where medical doctors recruited from CHUK and provincial hospitals were 4.8 times more likely to have good peri‐operative practices compared to those recruited from district hospitals and medicalized health centers (OR = 4.81, *p* = 0.016).

## 4 Discussion

This study provides a detailed examination of practice and adherence to operative strategies for CD in accordance with the WHO safe surgery checklist for CS among doctors in Rwanda. Our findings indicate there are significant gaps in pre‐, intra‐, and post‐operative practices among physicians who practice CS in Rwanda, which is dictated by level of experience and physicians’ job titles.

A notable finding is the lack of adherence to pre‐operative protocols, with 78.26% of participants not conducting team handovers or using the WHO SSC. This is consistent with studies highlighting the importance of these protocols in reducing surgical complications. For instance, studies have demonstrated that pre‐operative team briefings and adherence to safety checklists are crucial for improving surgical outcomes and reducing errors [11, 12, 13]. The absence of these practices in our study reflects a broader issue observed in low‐resource settings where adherence to standardized safety protocols often lags behind more developed healthcare systems [14]. The significant proportion of practitioners (84.78%) not ensuring pre‐cesarean patient bathing with soap contradicts the best practices recommended by the WHO [15], though the use of chlorhexidine for abdominal skin preparation (97.8%) aligns with recommended practices. The findings from our study underscore the need for improved adherence to infection control measures to enhance patient safety.

Studies from developed countries have shown that the implementation of such prophylactic measures is critical in preventing complications like deep vein thrombosis [16, 17]. The finding that 97.8% of medical doctors do not perform intraoperative hair removal during CD indicates a high adherence to modern evidence‐based guidelines that recommend avoiding unnecessary hair removal to reduce the risk of SSIs, reflecting effective dissemination of best practices and possibly contributing to improved patient safety and resource utilization within the healthcare system, and this is in accordance with the current literature [18, 19]. The high rate of regional anesthesia use (97.83%) and the near‐universal application of oxytocin or carbetocin (97.8%) for uterine contraction management are positive aspects, reflecting adherence to effective practices for pain management and hemorrhage prevention during CSs [20].

Among the interesting findings of our study are the existence of significant variability in operative techniques. The use of the Pfannenstiel incision by 71.7% of practitioners is in line with global recommendations, as this technique is preferred for its advantages in reducing post‐operative pain and facilitating recovery [21]. However, the practice of double‐layered hysterotomy closure (97.8%) is a positive indicator, given its association with reduced post‐operative complications [22]. The finding that 71.7% of practitioners correctly place the hysterotomy site approximately 6 cm above the pubic bone reveals a critical gap in CD practices. Although a majority adhere to the recommended anatomical guidelines, nearly 30% perform the incision incorrectly, which can lead to increased risk of complications such as injury to adjacent structures, wound infections, and prolonged hospital stays. Incorrect incision placement may also impact neonatal outcomes and overall surgical effectiveness. This highlights the need for improved training and adherence to surgical protocols to ensure all practitioners consistently apply best practices, thereby reducing complications and enhancing patient safety. The finding of 71.74% of practitioners use controlled cord traction followed by a manual sweep for placental delivery, whereas 28.3% use controlled cord traction alone, underscores a significant divergence in practices. According to established guidelines, the manual sweep should not be performed after placenta delivery, as it may lead to complications such as retained placenta, uterine trauma, or excessive bleeding [23]. Manual sweep, which involves manually assisting in the separation of the placenta from the uterine wall, is recommended only when necessary to ensure complete placental removal and prevent hemorrhage. The fact that a substantial proportion of practitioners are using manual sweeps incorrectly suggests a need for better adherence to clinical guidelines and improved training.

Although the finding of lack of compliance with WHO recommendations in our study context is alarmingly high, reflecting inadequate training and personnel to fulfill the clinical workload, it supports that geographical variability exists in obstetric surgical practices across countries worldwide [24]. This underscores the key role that professional organizations (such as WHO, FIGO, and Rwandan Society of Obstetricians and Gynecologists) play in order to have standardization of measures and guidance.

## 5 Conclusion

Results of this study show significant gaps in pre‐operative, intra‐operative, and post‐operative practices of WHO safe surgery checklist(SSC) for Cesearan delivery in Rwanda. Notably, a high proportion of practitioners do not follow essential pre‐operative protocols, such as team handovers and there is limited adherence to infection control practices like pre‐operative patient bathing with soap and vaginal preparation. We recommend uniform implementation of mandatory Cesearan delivery  protocols that adopt the WHO SSC. This implementation of clinical protocols may improve the quality of care provided; however, oversight by scientific and professional organizations such as the Rwandan Society of Obstetricians and Gynecologists(RSOG) is necessary for sustained change in good clinical practice across Rwanda.

## Author Contributions

Rusagura Rwizigira Eric and Diomede Ntasumbumuyange contributed to the conception and development of the study design, data collection, and data analysis. Diomede Ntasumbumuyange, Rusagura Rwizigira Eric, and Abraham Fessehaye Sium contributed to the manuscript write‐up. The final manuscript was edited by Abraham Fessehaye Sium. All authors critically revised the article for intellectual content. All authors reviewed the final manuscript and approved its submission.

## Disclosure

Abraham Fessehaye Sium is an editorial board member of Public Health Challenges (Wiley), and he was excluded for the editorial decision for this article.

## Ethics Statement

The study protocol was reviewed and approved by the Institutional Review Board of the College of Medicine and Health Sciences of the University of Rwanda. The principal investigator ensured the confidentiality and anonymity of the study participants by assigning unique code numbers to each participant.

## Consent

Participants had signed a written informed consent form prior to taking part in the study.

## Conflicts of Interest

The authors declare no conflicts of interest.

## Data Availability

The data that support the findings of this study are available on request from the corresponding author. The data are not publicly available due to privacy or ethical restrictions.
